# Impact of Biological Sex on Emotional Perception Among Adults With Schizophrenia Spectrum Disorders: Protocol for a Systematic Review

**DOI:** 10.2196/56977

**Published:** 2024-09-10

**Authors:** Gabrielle Ritchie, Harriet Nwachukwu, Stephen Parker, Frances Dark

**Affiliations:** 1 Metro South Addiction and Mental Health Services Woolloongabba Australia; 2 Faculty of Medicine The University of Queensland Brisbane Australia; 3 Metro North Mental Health Services Herston Australia

**Keywords:** schizophrenia, emotional perception, systematic review, sex differences, psychosis, social cognition, emotional processing

## Abstract

**Background:**

It is well established that individuals with schizophrenia experience deficits in emotional perception that can impact long-term social and occupational functioning. Understanding the factors that impact these impairments is important for targeting interventions to improve recovery. In the general population, compared with males, females tend to show greater perception of emotions. Whether this sex difference persists in schizophrenia is less clear. In contrast to males, females diagnosed with schizophrenia tend to have a higher age of disease onset and better premorbid functioning but do not necessarily have better outcomes. Effective treatments for social cognitive impairments are highly relevant to long-term functional rehabilitation. A greater understanding of the cognitive deficits in emotional perception within females and males living with schizophrenia may assist interventions to be better tailored to individuals.

**Objective:**

This systematic review aims to collate, synthesize, and critically appraise evidence considering the influence of biological sex (female and male) on the emotional perception of individuals with schizophrenia.

**Methods:**

This is a systematic review protocol based on the PRISMA-P (Preferred Reporting Items for Systematic Reviews and Meta-Analyses Protocols) guidelines. The electronic databases MEDLINE, Embase, CENTRAL, CINAHL, and PsycINFO will be systematically searched. To be included in this review, studies must compare the emotional perceptions of male and female participants older than 18 years who have a primary diagnosis of a schizophrenia spectrum disorder. Qualitative studies, case reports, case series, unpublished manuscripts, and studies not reported in English will be excluded. Key search strategies will include combinations of the following terms: “men,” “male,” “man,” “female,” “women,” “woman,” “sex,” “gender,” “emotional perception,” “emotional processing,” “schizophrenia,” “schizophren,” “psychotic disorders,” “psychosis,” “psychoses,” “psychotic,” “schizoaffective,” “schizotypal personality disorder,” and “schizotyp.” Identified studies will be uploaded to the web-based Covidence systematic review management software. The risk of bias for individual studies will be assessed using the relevant Joanna Briggs Institute checklist tools. The GRADE (Grading of Recommendations Assessment, Development, and Evaluation) system will also be used to evaluate the strength of the evidence base. Findings will be synthesized to provide a systematic summary of the existing literature. If sufficiently comparable data to permit meta-analysis emerges, a random-effects meta-analysis will be performed.

**Results:**

This systematic review was registered with the PROSPERO (International Prospective Register of Systematic Reviews) in October 2023. The search and screening of study titles and abstracts are currently underway. Data are expected to be extracted and analyzed in July 2024.

**Conclusions:**

Results will contribute to an improved understanding of the social cognitive profiles of males and females with schizophrenia. This knowledge is expected to inform the adaptation of interventions to improve functional outcomes.

**Trial Registration:**

PROSPERO CRD42023463561; https://tinyurl.com/34sr3rnf

**International Registered Report Identifier (IRRID):**

DERR1-10.2196/56977

## Introduction

Schizophrenia spectrum disorders (SSDs) are estimated to affect 24 million (or 1 in 300) people globally [1]. Overall mortality rates for people with schizophrenia are 3 times greater than for the general population [2], with life expectancy reduced by an average of 14.5 years [3]. These disorders are characterized by both positive (ie, hallucinations, delusions, and disorganized thoughts and behavior) and negative (ie, reduced speech and emotional expression, social withdrawal, and anhedonia) symptoms [4], which can contribute to significant distress and functional impairment across multiple life domains. Deficits in cognition are also a key feature of SSD, and their presence is predictive of poor functional outcomes [5,6]. Current pharmacological treatments have relative success in reducing positive psychiatric symptoms; however, they have limited capacity to target cognitive impairments [7,8]. These cognitive deficits extend not only to neurocognitive domains (eg, memory and attention) but also to social cognitive processes (ie, those involved in social interactions) [9-12]. In particular, it is well established that individuals with SSD have problems with emotional perception [13], the mental operations involved in the ability to understand and recognize the emotions of others [9].

There is strong evidence that individuals with schizophrenia perform significantly worse on tasks of emotional perception when compared with healthy individuals [13,14]. These deficits have been found to persist across different stages of the illness, worsen with the number of psychotic episodes, and be present in those at risk of developing schizophrenia [15-17]. It is noteworthy that previous meta-analyses of emotional perception in schizophrenia have tended to focus on facial emotion perception (FEP) [14]. This is unsurprising given that emotional perception is often conceptualized as the information conveyed in facial expressions. However, it can include other nonverbal (eg, body posture, movement, and physical touch) and verbal communication cues (eg, speech rate, vocal pitch, and voice intensity) [18]. Meta-analyses that have looked at the recognition of emotional perception in speech [19,20] have demonstrated significant deficits in individuals with schizophrenia, suggesting that emotional perception impairments extend across both verbal and nonverbal channels of communication. These impairments in emotional perception mean that individuals with schizophrenia can find adjusting and responding to social situations more challenging than healthy individuals. This can negatively impact a range of functional outcomes, including the ability to work and live independently [21], as well as the ability to demonstrate effective social skills and social problem-solving [22], both of which are important for developing meaningful social connections and support.

Despite emotional perception deficits being well established in the literature, there is still a lack of consensus regarding the specific factors contributing to impairments in this social cognitive domain [9]. For instance, in nonclinical populations, there appears to be a relationship between emotional perception and sex, such that females tend to perceive emotions better than males [23-25]. However, it is unclear whether this female advantage exists in schizophrenia [26-29]. Females with schizophrenia typically present with disease onset later than males and thus have higher premorbid functioning, although not necessarily better long-term outcomes [30-32]. There are a range of sex-related differences in the prevalence, presentation, and management of schizophrenia; however, these differences are still poorly understood, and research in this field is still in its infancy [33]. We identified 1 systematic review and meta-analysis [34] on the sex differences in FEP in a schizophrenia population. The sample included adults with chronic schizophrenia, individuals with early or recent onset of schizophrenia, and individuals with a first-degree relative with schizophrenia. This review found limited studies that reported sex differences in FEP, although those that did tended to show that females performed better than males. This study’s authors emphasized the importance of future research into the potential sex differences in FEP to improve the long-term outcomes of individuals with schizophrenia. Indeed, biomedical research, more generally, has tended to demonstrate sex disparity in participant recruitment (ie, males favored over females) [35]; there is a need for more research into sex-related differences in SSD [33]. Indeed, the inclusion of sex-specific clinical practice guidelines to guide the treatment of schizophrenia has been considered [35] as a potential step in managing the disorder.

Social cognition is significantly associated with long-term function, irrespective of factors such as symptom severity and length of illness [36]. It can also act as a mediator between neurocognition and functional outcomes [5,37]. Effective treatments for social cognitive impairments are highly relevant to long-term functional rehabilitation. We argue that a greater understanding of the deficits in emotional perception, a key aspect of social cognition, is needed to assist such interventions to be better tailored to individuals with schizophrenia. We also argue that understanding the role individual variables, such as biological sex, play on emotional perception task performance is important in developing the social cognitive profile of schizophrenia, particularly for females with the disorder, that has typically been underresearched. Previous systematic reviews and meta-analyses have studied emotional perception impairments in schizophrenia; however, preliminary searches in the PROSPERO (International Prospective Register of Systematic Reviews), CENTRAL, and the Joanna Briggs Institute Database of Systematic Reviews revealed no recent systematic reviews that have examined the role of biological sex on this social cognitive domain and no previous systematic reviews that have looked at the impact of biological sex on emotional perception outside of FEP tasks. This study aims to systematically review and critically appraise the quantitative literature considering the impact of biological sex on emotional perception in adults with SSDs. Our primary goal is to identify differences in emotional perception performance between males and females to contribute to the knowledge of the social cognitive profile of schizophrenia.

## Methods

This protocol was developed in consultation with the PRISMA-P (Preferred Reporting Items for Systematic Reviews and Meta-Analyses Protocols) guidelines [38,39]. This systematic review is registered with PROSPERO (ID: PROSPERO 2023 CRD42023463561).

### Eligibility Criteria

#### Participants

Participants must be older than 18 years with a primary diagnosis of SSD, as diagnosed by any recognized diagnostic criteria, such as the *DSM-V* (*Diagnostic and Statistical Manual of Mental Disorders* [Fifth Edition]) or the *ICD-11* (*International Classification of Diseases, 11th Revision*). Studies that include animals, children, or adolescents will be excluded. Participants who are at high risk of schizophrenia or who have experienced a first episode of psychosis but do not yet meet the full criteria for an SSD diagnosis will be excluded from this review. Studies that group adolescents and young adults together will also be excluded unless it is possible to separate participants who are older than 18 years.

#### Intervention

Studies will not need to include an intervention to be included in this systematic review. However, studies will need to examine at least 1 measure of emotional perception (eg, Bell Lysaker Emotion Recognition Task [28]; Penn Emotion Recognition Test [29]). We will define emotional perception as the social cognitive processes involved in comprehending and recognizing emotions in others. We will include any aspect of emotional perception (ie, verbal, and visual). Studies that are attempting to validate new tasks will be excluded.

#### Comparator

There will be no restriction on the inclusion of comparator groups (eg, intervention, treatment-as-usual, and healthy controls); however, studies must include male and female participants. Studies that combine participants (both males and females) into 1 group can also be included if the available data allow us to separate these participant groups. Studies that do not report the biological sex of participants will be excluded.

#### Outcomes

This systematic review will include quantitative, empirical studies with no restrictions on the type of setting or timing of publication. The exclusion criteria include qualitative studies, case reports, case series, unpublished manuscripts or abstracts, and papers not available in English. Studies must provide performance outcomes on the emotional perception task. These may be primary or secondary outcomes of the included studies.

### Search Strategy

Initial search terms will be developed and piloted by the research team. Key search terms will include “men,” “male,” “man,” “female,” “women,” “woman,” “sex,” “gender,” “emotional perception,” “emotional processing,” “schizophrenia,” “schizophren,” “psychotic disorders,” “psychosis,” “psychoses,” “psychotic,” “schizoaffective,” “schizotypal personality disorder,” and “schizotyp.” Boolean operators “OR” and “AND” will combine these keywords. We will build a search terms and limits table to consider the keywords, terminology, and search limits. This table will also capture these keywords’ synonyms, abbreviations, alternative spellings, and collective nouns. We will use the MeSH (Medical Subject Headings) Browser to search for our topics and review the MeSH tree structures for each keyword to see whether there are any narrower terms to include in our search. We will then use this table to develop a preliminary search strategy with MEDLINE (PubMed; Multimedia Appendix 1). Peer review will be sought from a health services librarian with expertise in systematic reviews and meta-analyses. We will use the Polyglot search translator to adjust and translate our search strategy syntax to different databases [40]. The following electronic databases will be searched: MEDLINE, Embase, CENTRAL, CINAHL, and PsycINFO. Reference lists of included studies will also be manually scanned. Databases will continue to be searched for new publications until the end of June 2024. Study authors may be contacted to request full-text publications and clarify questions about study results. We will also search, at minimum, the first 3 pages of Google and Google Scholar to test our search strategy.

### Selection Process

The literature search results will be uploaded to the web-based Covidence systematic review management software (Veritas Health Innovation) [41]. Abstracts that meet the search terms will be imported into Covidence, and titles and abstracts will be screened to determine whether they meet the inclusion criteria for a full-text review. Any duplicates will be removed during the screening process. Full-text papers will be uploaded for the formal reviewing process. If full-text versions are unavailable, the researchers will attempt to contact the study authors to request a copy of their paper via a maximum of 3 email attempts. Researchers (FD and GR) will independently screen titles and abstracts based on the study selection criteria. Before this screening process, screening questions will be developed to assist reviewers. Covidence software will flag any conflicts between reviewers at each of these stages. Reviewers will meet to discuss and resolve these conflicts. A third reviewer (HN) will be sought if a consensus cannot be reached. Full-text versions of studies that are considered relevant will then be formally reviewed by at least 3 researchers. Again, any conflicts during this process will be flagged, and reviewers will meet to seek consensus. Another reviewer will be contacted if an agreement cannot be reached. Eligible full-text publications will be stored in Endnote referencing software using smart groups. These selected papers will be checked for retractions and errata. Before data extraction, a search will be rerun to identify any new studies to be retrieved for inclusion. A PRISMA (Preferred Reporting Items for Systematic Reviews and Meta-Analyses) flow diagram will record search results during the study selection process [42].

### Methodological Quality Assessment

Two reviewers will independently critically appraise the quality of studies included in the systematic review for risk of bias using the relevant Joanna Briggs Institute checklist tools [43]. These tools provide checklists for a range of study designs, including randomized and nonrandomized controlled trials. Assessment will consider potential sources of bias for each study, such as in the selection of participants, reliability in the measurement of outcomes, appropriateness of the statistical analysis undertaken, and potential selective reporting of results. Reviewers will meet to resolve any discrepancies in these ratings. A third reviewer will be sought if required. No reviewers will be blind to journal titles, study authors, or institutions. We will report the results of this quality assessment in tabular format in the systematic review.

### Data Extraction and Synthesis

One researcher will extract the data from the selected full-text publications using the web-based Covidence systematic review management software [41]. This process will be reviewed by a second researcher. A data extraction template will be developed, piloted, and reviewed by the researchers before formal data extraction. If any data are unclear, the researchers will attempt to contact study authors to clarify their findings via a maximum of 3 email attempts over 28 days. We expect to extract the following data from the selected full-text publications as available: author and institution details, publication information (including study title, journal title, publication date, and study location), participant demographic and clinical information (including age, biological sex, primary and secondary mental health diagnoses, mean duration of onset of symptoms, premorbid functioning, current stage of illness, any other cognitive or physical impairments, and current medications), study methodology (including trial design, trial size, and emotional perception task), and all quantitative data on dynamic perception task performance including any dropouts or missing data.

The characteristics of the included studies will be presented in tabular format and summarized in narrative synthesis as appropriate. If sufficiently comparable data to permit meta-analysis emerges, this will be completed using the random-effects model. Both continuous (eg, standardized mean difference) and categorical outcomes (eg, odds ratio and relative risk) in the extracted studies will be assessed and compared. The heterogeneity of effect sizes of pooled studies will be measured via forest plots using chi-square and *I*^2^ values. If appropriate, a funnel plot and Egger test will be used to report publication bias. No subgroup analyses are planned at this stage. The GRADE (Grading of Recommendations Assessment, Development, and Evaluation) system will be used to consider the strength of the evidence available [44]. Two reviewers will rate their level of confidence (ie, very low, low, moderate, or high) in the quality of evidence for outcomes. A third reviewer will be sought if consensus cannot be reached in these ratings. We will report this evaluation of the evidence in tabular format in the systematic review.

### Ethical Considerations

This study does not require ethics approval and consent to participate. No data used for analysis or reported in the systematic review will be identifiable.

## Results

This systematic review was submitted to PROSPERO in September 2023 and registered in October 2023. As of January 2024, preliminary searches and piloting of the study selection process have been completed. Formal screening of study titles and abstracts against eligibility criteria has commenced, with 973 studies imported to Covidence management software for screening. Data are expected to be extracted and analyzed in July 2024, and we aim to submit results for peer-reviewed publication by December 2024. This study is self-funded.

## Discussion

### Principal Findings

This study will systematically review the impact of biological sex on emotional perception in adults with SSD. In comparison with the general population, deficits in emotional perception in schizophrenia are well established [13,14], but there is very little research into the potential impact sex has on performance. We did identify 1 systematic review and meta-analysis [34] that found only limited evidence of a female advantage for emotional perception. However, it is important to note that this construct was restricted to FEP tasks, and the study authors highlighted the need for future research into this area. We hypothesize that our review may provide support to these findings. In healthy adults, females tend to perform better at emotional perception tasks. Our review aims to understand whether this advantage extends to females with schizophrenia or whether the advantage is attenuated. The social cognitive profile of schizophrenia, more generally, is still largely heterogeneous and how it differs to other psychiatric disorders is still under investigation [9]. We hope to provide a greater understanding of how sex-related differences in emotional perception may explain some of this variation and, in turn, inform future treatment that targets functional outcomes in these individuals. The need for more research into sex-related differences in schizophrenia has been emphasized as a new but important field of research [33].

### Limitations

The main limitation anticipated in this review relates to the lack of consensus within the literature on how to measure emotional perception [12], compounded by the limited availability of well-validated and reliable tools. Often, attempts have been made to adapt measures from other participant samples to a schizophrenia population but with limited research on the psychometric properties of these measures in SSD [9]. A similar critique relates to existing social cognitive tasks’ lack of cross-cultural validity [9]. These restraints may limit the ability to draw conclusions and pool outcomes from comparable studies. If this is the case, we plan to provide a narrative synthesis and group studies by the type of emotional perception task, reporting on the reliability and validity of these measures. The study researchers must also acknowledge the cultural biases we bring to this review and be cautious in generalizing any conclusions drawn to other cultures, especially given the limit to studies available in English included in our study criteria. We also plan to measure the quality of evidence to assess the confidence in the available data and report these findings in the systematic review.

### Conclusions

This study will review the evidence for sex differences in emotional perception within SSD. The results will provide a greater understanding of the social cognitive profile of schizophrenia and, in turn, inform future interventions tailored to improve long-term rehabilitation.

## Appendix

Multimedia Appendix 1 Draft MEDLINE search—PubMed interface.

## Abbreviations

*DSM-V*: *Diagnostic and Statistical Manual of Mental Disorders (Fifth Edition)*
FEP: facial emotion perception
GRADE: Grading of Recommendations Assessment, Development, and Evaluation
*ICD-11*: *International Classification of Diseases, 11th Revision*
MeSH: Medical Subject Headings
PRISMA: Preferred Reporting Items for Systematic Reviews and Meta-Analyses
PRISMA-P: Preferred Reporting Items for Systematic Reviews and Meta-Analyses Protocol Statement
PROSPERO: International Prospective Register of Systematic Reviews
SSD: schizophrenia spectrum disorder

## Data Availability

Access to research data is restricted and governed by the Queensland Health Government; however, extracted data may be available upon request. All researchers will have access to the final data set. Extracted data will be made publicly available as multimedia appendices.
