# Supplementary material for: Impact of Biological Sex on Emotional Perception Among Adults With Schizophrenia Spectrum Disorders: Protocol for a Systematic Review
Source: JMIR Res Protoc. 2024 Sep 10;13:e56977. doi: 10.2196/56977 (PMC11422750; doi:10.2196/56977)
Supplement: Multimedia Appendix 1 [file resprot_v13i1e56977_app1.docx]

| Search number | Query | Results |
| --- | --- | --- |
| 4 | #1 AND #2 AND #3 | 306 |
| 3 | ("emotional perception"[ti:~3] OR "emotional processing"[ti:~3] OR "emotional recognition"[ti:~3] OR "emotional task"[ti:~3] OR "emotional test"[ti:~3] OR "emotional perception"[tiab] OR "emotional processing"[tiab] OR "emotional recognition"[tiab] OR "emotional task"[tiab] OR "emotional test"[tiab] OR "bell lysaker emotion recognition task"[tiab] OR "penn emotion recognition Test"[tiab] OR "social cognition psychometric"[tiab]) | 5,776 |
| 2 | (schizophren*[tiab] OR psychosis[tiab] OR psychoses[tiab] OR psychotic[tiab] OR schizoaffective[tiab] OR schizotyp*[tiab] OR schizophrenia[mh] OR "psychotic disorders"[mh] OR "schizotypal personality disorder"[mh]) | 179,149 |
| 1 | (gender[tiab] OR sex[tiab] OR male[tiab] OR males[tiab] OR men[tiab] OR mens[tiab] OR "men's"[tiab] OR man[tiab] OR female[tiab] OR females[tiab] OR woman*[tiab] OR women*[tiab] OR women[mh] OR men[mh] OR male[mh]) | 9,492,374 |

Filters: English, MEDLINE
